# Early expansive single sided laminoplasty decompression treatment severe traumatic cervical spinal cord injury

**DOI:** 10.3389/fsurg.2022.984899

**Published:** 2022-09-16

**Authors:** Chaohua Yang, Qing Wang, Shuang Xu, Can Guan, Guangzhou Li, Gaoju Wang

**Affiliations:** ^1^ Department of Orthopaedics, The Affiliated Hospital of Southwest Medical University, Luzhou, China; ^2^ Department of Orthopedic surgery, The First Affiliated Hospital of Chongqing Medical University, Chongqing, China; ^3^ Department of Orthopaedics, Xuanhan People's Hospital, DaZhou, China

**Keywords:** laminoplasty, expansive decompression, cervical spinal cord injury, intramedullary lesion length, early surgery

## Abstract

**Background:**

Severe traumatic cervical spinal cord injury (tcSCI) is a disastrous event for patients and families. Maximizing spinal cord function recovery has become the primary therapeutic goal. This study investigated the effect of early extensive posterior decompression on spinal cord function improvement after severe tcSCI.

**Methods:**

A retrospective review of 83 consecutive patients who underwent extensive open-door laminoplasty decompression within 24 h after severe tcSCI (American Spinal Injury Association (ASIA) impairment scale (AIS) grade A to C) between 2009 and 2017 at our institution was performed. The patient clinical and demographic data were collected. Neurological functional recovery was evaluated according to the Japanese Orthopaedic Association (JOA) score system, ASIA motor score (AMS) and AIS grade.

**Results:**

Among the 83 patients initially included, the baseline AIS grade was A in 12, B in 28, and C in 43. Twenty-three patients (27.7%) had a high cervical injury. Cervical spinal stenosis (CSS) was identified in 37 patients (44.6%). The mean intramedullary lesion length was 59.6 ± 20.4 mm preoperatively and 34.2 ± 13.3 mm postoperatively (*p* < 0.0001). At the final follow-up visit, an improvement of at least one and two AIS grades was found in 75 (90.4%) and 41 (49.4%) patients, respectively. 24 (64.9%) patients with an improvement of least two AIS grades had CSS. The mean AMS and JOA score were significantly improved at discharge and the final follow-up visit compared with on admission (*p* < 0.0001).

**Conclusions:**

Our results suggest that early expansive laminoplasty decompression may improve neurological outcomes after severe tcSCI, especially in patients with CSS. Larger and prospective controlled studies are needed to validate these findings.

## Background

The cervical spinal cord is the most frequently affected segment in traumatic spinal cord injury (SCI), potentially leading to quadriplegia, lifelong disabling neurological sequelae and even death (1). Studies have shown that SCI can be divided into primary and secondary injury according to different injury stages and pathophysiological mechanisms (2). The primary goal in treatment is to maximize the improvement of spinal cord function and the extent of spinal cord decompression, attenuate secondary injury, and stabilize the spine (3).

To date, the surgical approaches for the treatment of severe tcSCI, including posterior, anterior, and combined posterior and anterior surgical approaches, remain controversial. In previous clinical studies, most researchers preferred an anterior approach because of the cited advantages, including the direct anterior decompression of fragmented intervertebral discs, the reduction of dislocated fractures, minimal surgical trauma, and fewer complications (4–6). However, the extent of spinal cord decompression that can be achieved *via* an anterior approach is inadequate. Research shows that greater degrees of cord decompression are associated with greater degrees of neurological recovery (7). Therefore, extensive posterior decompression (by laminoplasty or laminectomy) and reduction have gained increased attention from clinical researchers over recent years, with results including successful decompression, reduction for zygapophysis interlocking, and better alignment and motor unit preservation (8–12). In addition, in cases of huge disc herniation, burst fracture involving the vertebral posterior wall, and cervical kyphosis, the combined posterior and anterior approach is suitable, with the posterior approach allowing for stabilization and decompression, and the anterior approach for decompression disc and fracture fragments, and correction of kyphosis (13, 14). In this article, we examine the effect of extensive posterior open-door laminoplasty decompression within 24 h after severe tcSCI on spinal cord function improvement and investigate whether extensive decompression can promote the resolution of spinal cord edema.

## Patients and methods

### General information

This single-center, longitudinal, retrospective study was conducted to investigated the effect of early extensive laminoplasty decompression on spinal cord function improvement after severe tcSCI. The present study was approved by the Institutional Ethics Committee (ethics approval number: ky2018106) and informed consent was obtained from all patients. A cohort of 83 patients with severe tcSCI (AIS grade A to C), who underwent extensive open-door laminoplasty decompression (with or without pedicle screw fixation) within 24 h after trauma between January 2009 and January 2017 were included in the study. The exclusion criteria were as follows: central cord syndrome; complicating traumatic brain injury; history of cervical spine surgery; klippel-Feil syndrome; ankylosing spondylitis; tumors of other tissues or organs; intolerance to posterior approaches due to a poor general condition.

An electronic medical database was used to collect patient clinical and demographic information, including age, gender, mechanism of injury, fractures or dislocations, CSS, patient date of injury to operation, SCI level, operative procedures, operative time, blood loss, hyponatremia (serum sodium concentration <135 mmol/L), hypotension (arterial systolic blood pressure <90 mmHg) and tracheotomy.

### Surgical technique

Decompression surgery was performed within 24 h following trauma. The operation program was implemented as follows: all patients underwent posterior open-door laminoplasty; in the patients with fractures and dislocations, pedicle screw fixation was performed simultaneously; a second stage anterior surgery was added when neurological deterioration occurred due to a large nonreduced disc fragment identified on preoperative magnetic resonance imaging (MRI). Following general anesthesia, the patient was placed in the prone position and their position was fixed using a Mayfield head holder, and continuous skull traction was performed with 5–8 kg weights to allow a maximally horizontal head position. The laminae were exposed through detached the bilateral paravertebral muscles, then the processes were removed. The range of laminoplasty decompression was from at least one lamina above and below the edematous segment according to MRI and/or absence of the subarachnoid space (SAS) (15). Then, single open-door laminoplasty was performed using a high-speed air-burr drill (15). Usually, the side with severely paralyzed as the door opening side, and the other side as the hinge side. Reduction and pedicle screw fixation was performed in patients with fractures and dislocations using a previously described method (8, 12, 15). To relieve spinal cord compression as soon as possible, we usually implemented open-door first, then inserted the pedicle screw to recovery cervical alignment, kept the door open with titanium plate or threads last.

### Postsurgical treatments

Conventional drainage under negative pressure was applied for 1–3 days after the operation. The drainage tube remained in place for 7–9 days when the patient complicated with cerebrospinal fluid (CSF) leakage. Antibiotics, dexamethasone, mannitol, and neurotrophic agents (ganglioside) were routinely used for 3–5 days after surgery. Patients were protected by a cervical collar for approximately 1 month postoperatively. The patients who were discharged to a rehabilitation center, the mean hospital length of stay were 17.2 ± 4.3 days.

### Imaging analysis

**Figure 1:**
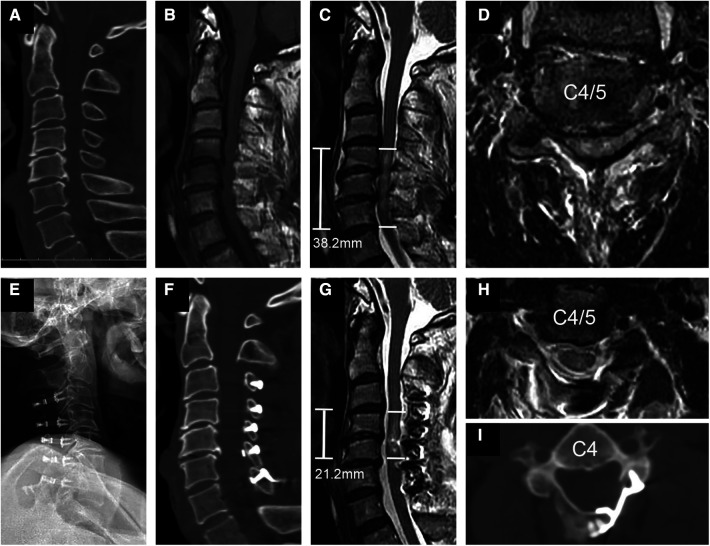
Representational preoperative (**A–D**) and postoperative (**E–I**) images of a 58-year-old male patient who sustained a spinal cord injury without fracture or dislocation. His AMS was 18 and AIS grade C. Midsagittal subaxial CT (**A**) indicated cervical spinal stenosis (CSS) from C3 to C6, and MRI (**B**) SCI with an intramedullary lesion length (IMLL) of 38.2 mm. The subarachnoid space (SAS) was absent at C4 and C5 (**C,D**). Laminoplasty of C3–7 was performed in this patient 17 h after trauma. Postoperative x-rays (**E**) and CT (**F,I**) showed significant enlargement of the osseous spinal canal. Postoperative MRI showed an IMLL of 21.2 mm (**G**) and successful decompression (**G,H**) indicated by the presence of an open anterior and posterior SAS 11 days after trauma. One year after injury, the patient recovered from paralysis with an AMS of 100 and AIS grade of E.

**Figure 2:**
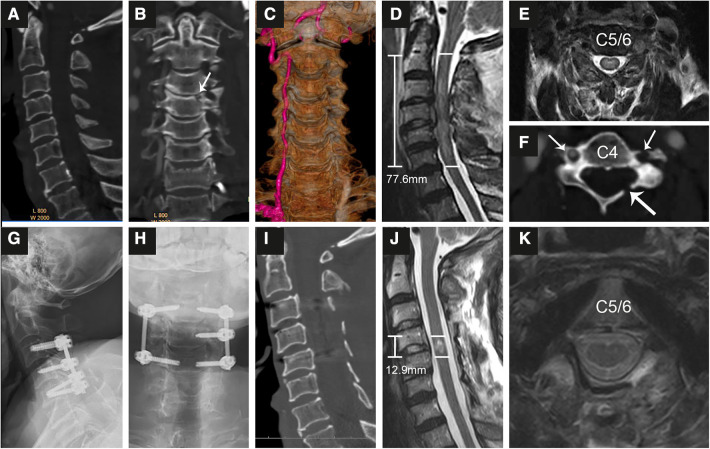
Representational preoperative (**A–F**) and postoperative (**G–K**) images of a 68-year-old male patient who sustained an A1 type fracture and spinal cord injury. His AMS was 11 and AIS grade B. Midsagittal (**A**), coronal (**B,C**) and axial (**F**) enhanced CT indicated an A1 type fracture at C4 (**B**) accompanied by fractures of the left lamina (**F**, long arrow) and transverse foramen (short arrow) and injury to the left vertebral artery (**C**). MRI (**D,E**) showed SCI with an intramedullary lesion length (IMLL) of 77.6 mm. The subarachnoid space (SAS) was absent from C3 to C5 (**D**). Laminoplasty of C3–7 and pedicle screw fixation from C3–C5 were performed in this patient 9 h after trauma. Postoperative x-rays (**G,H**) and CT (**I**) showed good screw positioning and significant enlargement of the osseous spinal canal. Postoperative MRI indicated an IMLL of 12.9 mm (**J**), successful decompression (**J**), and reduced edema 12 days after trauma. One year after injury, the patient paralysis recovery with an AMS of 72 and AIS grade **D**.

**Figure 3:**
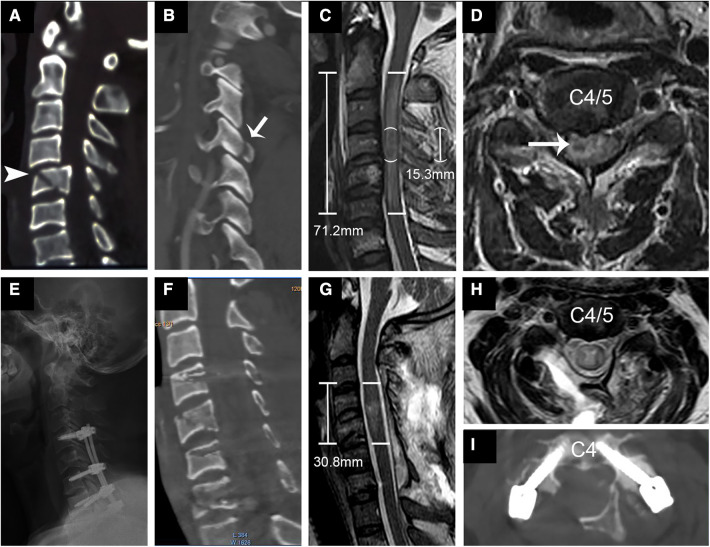
Representational preoperative (**A–D**) and postoperative (**E–I**) images of a 44-year-old male patient who sustained a C type fracture and spinal cord injury. His AMS was 3 and AIS grade A. Sagittal subaxial CT indicated C4 translation rotation injury (**A**) accompanied by fracture of the right inferior articular process (**B**, short arrow) and C5 teardrop fracture (arrowhead). MRI (**C,D**) showed SCI with an IMLL of 71.2 mm and intramedullary hemorrhage (long arrow) 15.3 mm in length. The subarachnoid space (SAS) was absent from C3 to C5 (**C**). Laminoplasty of C3–7 and pedicle screw fixation from C4–C7 were performed in this patient 14 h after trauma. Postoperative x-rays (**E**) and CT (**F,I**) showed good screw positioning and significant enlargement of the osseous spinal canal. Postoperative MRI indicated an IMLL of 30.8 mm (**G**) and successful decompression (**G,H**) 12 days after trauma. One year after injury, the patient paralysis recovery with an AMS of 58 and AIS grade **C**.

Pre- and postoperative and follow-up imaging studies were conducted for patients, and the measurement results were evaluated by two independent, blinded spine surgeons and one imaging diagnostician. X-ray and computed tomography (CT) images were used to measure the Torg–Pavlov ratio [TPR; TPR less than 0.82 indicates cervical spinal stenosis (CSS)] (16) and to classify the injury morphology according to the AOSpine subaxial cervical spine injury classification system preoperatively (17). Additionally, radiological examination of the cervical spine was performed postoperatively for patients to determine the exact surgical procedure, cervical anatomical alignment, and degree of bony decompression. MRI of the cervical spine was performed using a 1.5-Tesla Siemens MRI system (Siemens Magnetom; Aera, Erlangen, Germany). Preoperative MRI data were available for review in 95.2% of cases, obtained within 9.3 ± 6.8 h after trauma; postoperative MRI data were available for review in 90.4% of cases, obtained at 10.8 ± 0.95 days after trauma. Quantitative and qualitative measurements obtained for this study included the intramedullary lesion length (IMLL), hematoma length, decompression, injury and intervertebral disc sequestration, as previously described (Figures 1, 2, 3) (18). Successful decompression was defined as the patency of CSF pathways and the presence of an open SAS around a contused and swollen spinal cord (Figures 1G, 2J, 3G).

### Clinical assessment

Neurological functional recovery and grade conversion were evaluated according to the ASIA motor score (AMS) and impairment scale (AIS) grade on admission, at hospital discharge, and at the final follow-up visit (19). In addition, Japanese Orthopaedic Association (JOA) score (20) was recorded at the same time points. The recovery rate was calculated using the method described by Hirabayashi to compare the pre- and postoperative JOA scores (21). Patients were followed for at least 1 year (3.7 ± 2.4 years) after trauma.

### Statistical analysis

Statistical data analysis was performed using SPSS software version 19.0 (IBM, New York, NY, United States). All data were expressed as mean ± standard deviation (SD). Independent samples t test was used for the statistical analysis of parametrically distributed variables. Fisher's exact test and chi-squared test were used for categorical variables. The Manne-Whitney *U* test was used to evaluate the association of postoperative scores (AMS, JOA score). *p* < 0.05 was considered statistically significant.

## Results

### Clinical characteristics

**Figure 4:**
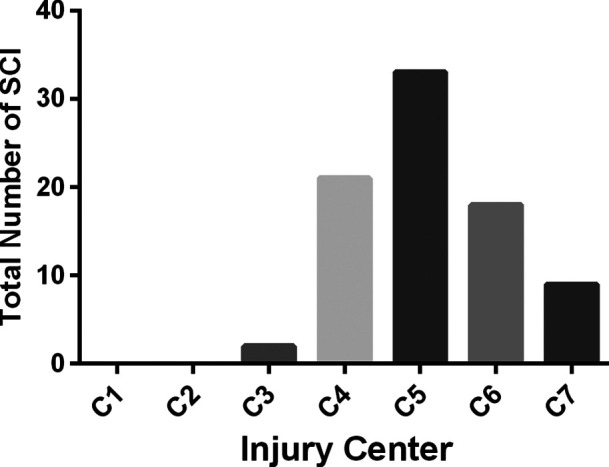
Number of spinal cord injuries at various levels.

A total of 83 patients (male: 76; female: 7) met the eligibility criteria. The key demographic, clinical, and outcome parameters of these patients are summarized in Table 1. Among the 83 patients initially included in the trial, the baseline AIS grade was A in 12, B in 28, and C in 43. The level of SCI is shown in Figure 4; of the patients, 23 had a high cervical injury (C1 to C4, 27.7%). Of the 83 patients, 8 (9.6%) underwent four-level laminoplasty, 73 underwent five-level laminoplasty, and 2 underwent six-level laminoplasty. In addition to the laminoplasty, an additional dorsal spinal stabilization was performed in 29 cases. No patients required an anterior surgical intervention for additional decompression of a large, nonreduced disc fragment.

**Table 1:** Demographic and clinical characteristics of patients.

Characteristic	Value
Number of patients	83
Male-to-female ratio	76:7
Age, years, mean ± SD	54.3 ± 10.9
Duration trauma to MRI, hours, mean ± SD	9.3 ± 6.8
Duration trauma to surgery, hours, mean ± SD	16.5 ± 7.1
**AOSpine fractures classification**	
Type A0	54
Type B2	10
Type B3	7
Type C	12
Cervical spinal stenosis (%)	37 (44.6)
**Injury mechanism**	
Traffic accidents (%)	17 (20.5)
Fall injuries (%)	55 (66.3)
Heavy load injuries (%)	11 (13.3)
**AIS Grade**	
Grade A (%)	12 (14.5)
Grade B (%)	28 (33.7)
Grade C (%)	43 (51.8)
**Level of Laminoplasty**	
Laminoplasty of four-level (%)	8 (9.6)
Laminoplasty of five-level (%)	73 (88.0)
Laminoplasty of six-level (%)	2 (2.4)
Dorsal spinal stabilization (%)	29 (34.9)
Surgical duration, minutes, mean ± SD	92 ± 43
Blood loss, mL, mean ± SD	173 ± 76
Time of hospital stay, days, mean ± SD	17.2 ± 4.3
Time of follow-up, years, mean ± SD	3.7 ± 2.4

### Postoperative complications

Dural leakage was observed in 7 (8.4%) cases after surgery and treated by pressure suture drainage orifice after 7–9 days of drainage. Two patients had incision infection and one had central nervous system infection. They were cured by changing dressings, using sensitive antibiotics and lumbar cistern drainage. Six patients developed pneumonia. The incidence of hyponatremia, hypotension and tracheotomy was 59%, 14.5% and 3.6%, respectively. A follow-up period of least 1 year was achieved for 77 patients, with a retention rate of 92.8%. Six patients were lost to follow-up in the acute stage of SCI due to the following reasons: brain death in 1 case; respiratory failure in 3 cases; hypotension and cardiac arrest in 2 cases.

### Imaging results

Several studies have provided ample evidence that MRI scanning is useful for providing objective measures of tSCI to both distinguish the injury severity and predict the AIS grade conversion (18). All 79 subjects with MRI records were found to have spinal cord edema on MRI at admission (Table 2). The mean pre- and postoperative IMLL was 59.6 ± 20.4 mm and 34.2 ± 13.3 mm, respectively (*p* < 0.0001). Significant decreases in edema, as indicated by the signal intensity on T2 MRI, were observed far from the damage center postoperatively compared with preoperatively. No patients showed an increased IMLL or complete fading of the edema on MRI after surgery. Intramedullary spinal cord hemorrhage was demonstrated in 20.5% of patients, mostly in AIS grade A and B patients (Figure 3). The mean rostrocaudal hemorrhage length pre- and postoperatively was 5.11 ± 1.64 mm and 4.62 ± 1.41 mm, respectively (*p* = 0.55, Table 2). In 81 of 83 patients (97.6%), complete spinal cord decompression was achieved. The remaining two patients, who underwent C3–C7 laminoplasty, showed insufficient decompression due to large disc herniation and cervical spine sequence problems (anterior decompression was not performed due to significant improvement in spinal cord function).

**Table 2:** Mean intramedullary lesion length and hematoma length.

Variable	Preoperative	Postoperative	*t* value	*p* value
IMLL	59.6 ± 20.4	34.2 ± 13.3	9.46	*p* < 0.0001
Hemorrhage length	5.11 ± 1.64	4.62 ± 1.41	0.94	*p* = 0.55

Values are mean ± SD, mm. IMLL, intramedullary lesion length.

During the follow-up period, the injury sites were observed to be occupied by cysts and myelomalacia, as depicted by bright signals on T2 MRI. However, no syringomyelia or spinal atrophy was found on follow-up MRI. In all patients treated with a posterior approach, “close the door” was not observed after single open-door decompression. Loosening, dislocation, and breakage of the internal fixation instrumentation were not observed by radiological examination at the follow-up visit. Two patients showed cervical kyphosis during the follow-up period.

### Neurological outcomes

In the study group, neurological improvement according to the change in the AIS grade from the preoperative assessment to the final follow-up visit is represented in Table 3. No patients showed a worsening AIS grade at postoperatively. An improvement of at least two AIS grades and of at least one AIS grade was found in 41 (49.4%) and 75 (90.4%) patients at the final follow-up visit, respectively; 24 (64.9%) patients with an improvement of least two AIS grades had CSS. In some patients with CSS and diffuse hyperintense or faint signals on MRI, neurological function was significantly improved several hours after decompression.

**Table 3:** Changes in American spinal injury association impairment scale (AIS) grade from pre-operative to final follow-up.

Preoperative AIS grade	A	B	C	D	E	Total
A	3	1	6	2		12
B		5	4	17	2	28
C				29	14	43

The mean AMS on admission, at discharge and at the final follow-up visit was 24.5 ± 16.7, 49.4 ± 26.8 and 70.8 ± 23.0, respectively (Table 4). The AMS showed a significant improvement at discharge and at the final follow-up visit compared with that on admission (*p* < 0.001). The average JOA score was 1.49 ± 1.61 points on admission, 4.95 ± 3.44 points at discharge, and 10.98 ± 4.72 points at the final follow-up visit (Table 4). A significant improvement in the JOA score was achieved at discharge and at the final follow-up visit compared with that on admission (*p *< 0.001). The average recovery rate of the JOA score was 56.1 ± 23.1% at the final follow-up visit.

**Table 4:** AMS and JOA scores at admission, discharge and follow-up.

Scores	Admission	Discharge	Final follow-up
AMS	24.5 ± 16.7	49.4 ± 26.8*	70.8 ± 23.0*
JOA scores	1.49 ± 1.61	4.95 ± 3.44*	10.98 ± 4.72*

Values are mean ± SD.

AMS, American spinal injury association motor score; JOA, Japanese orthopaedic association.

*Significant differences for the discharge and final follow-up versus admission (*p < 0.001).

## Discussion

We performed a retrospective study at a single trauma center to evaluate the effect of extensive posterior decompression within 24 h after severe tcSCI on neurological outcomes. In accordance with our hypothesis, we found a significant and rapid improvement in neurological function in patients undergoing early extensive decompression by laminoplasty through the rapid resolution of spinal cord edema visualized *via* T2 MRI. An improvement of at least one and two AIS grades was found in 90.4% and 49.4% patients, respectively.

Numerous previous clinical studies on tcSCI have suggested that early surgical decompression might promote neurological recovery (22–26). In a multicenter study, Fehlings et al. demonstrated that early surgery (<24 h) resulted in superior neurological recovery at 6 months compared to late surgery (≥24 h) in patients with cervical SCI (27). In addition, Wilson et al. comparing the effect of early surgical decompression within 24 h to later time frames, they found that decompression before 24 h after tSCI was associated with significantly improved neurological outcomes (27, 28). In our study, we included 83 patients with tcSCI who underwent decompression within 24 h. The average AMS and JOA score was 70.8 ± 23.0 and 10.98 ± 4.72 points at the final follow-up visit compared with 24.5 ± 16.7 and 1.49 ± 1.61 points on admission, respectively (Table 4). This notable neurological recovery is consistent with previous studies. This result might be explained by early surgical decompression expeditiously relieving mechanical spinal cord compression, thereby improving the spinal cord blood supply to avoid or mitigate secondary damage cascades and SCHS and to facilitate the restoration of spinal cord function (2, 22, 29). Badhiwala and colleagues stated that “time is spine” (30) and highlighted that there is a critical time window after primary injury to the spinal cord during which secondary injury mechanisms, which cause further neural tissue destruction, may be curtailed (31).

Furthermore, Jug et al. demonstrated that patients with tcSCI who undergo surgical decompression within 8 h after injury have superior neurological outcomes than patients who undergo decompression 8–24 h after injury, without any increase in the rate of adverse effects (32). Among 22 patients (19 AIS grade A or B) who underwent decompression within the first 8 h after tcSCI, they found an improvement of at least one and two AIS grades in 72.7% and 45.5% patients, respectively, at 6 months (32). In the current study, an improvement of at least one and two AIS grades was observed in 90.4% and 49.4% patients at the final follow-up visit. The higher odds of achieving an improvement in the AIS grade of at least a one grade in our study than in Jug's study might be due to the difference in the surgical method used for decompression; extensive posterior laminoplasty (assisted with posterior pedicle screw fixation if necessary) was performed in our study, whereas anterior discectomy (ADF) or corpectomy (ACF) and fusion was preferred in Jug's study.

Obviously, more adequate decompression increases the possibility of upward AIS grade conversion (2, 7, 18). In a recent study, Piazza reported that posterior cervical laminectomy results in better radiological decompression of the posterior CSF space than does ADF (11). Unfortunately, the authors did not evaluate the clinical effect. Another study, by Aarabi, demonstrated that the rate of decompression in patients who underwent ADF and ACF without laminectomy was 46.8% and 58.6%, but that in patients who underwent laminectomy at one, two, three, four, and five levels was 58.3%, 68%, 78%, 80%, and 100%, respectively (9). They indicated that performing laminectomy for motor complete tcSCI patients significantly increased the rate of successful spinal cord decompression, leading to better neurological outcomes, independent of whether anterior surgery was performed (9). Additionally, recent therapeutic trials have confirmed the continued importance on the combined effects of the timing of surgical intervention and extent of surgical decompression on the tcSCI (33–35). Therefore, in this study, we implemented extensive laminoplasty within 24 h for severe tcSCI patients and found a significant increase in the rate of AIS grade conversion (90.4%) and improved neurological function, as determined by the AMS and JOA score (Table 4). Furthermore, MRI showed that the IMLL was significantly decreased 8–14 days (34.2 ± 13.3 mm) after surgery compared with preoperatively (59.6 ± 20.4 mm), and 97.6% of patients achieved complete decompression. This study indicates that combined early and extensive surgical decompression significantly promotes the recovery of spinal cord function through the rapid resolution of spinal edema.

On the other hand, it is possible that the superior rate of AIS grade conversion in our study compared with that in Jug's (32) study is because of the larger proportion of AIS grade C patients, who may be able to achieve better neurological recovery than AIS grade A and B patients (36, 37). In addition, we found that tcSCI patients without fractures or dislocations but with CSS could achieve significant neurological function recovery within several hours after extensive decompression, with a significantly higher rate of an improvement of two AIS grades than the others patients. This difference may be due to early extensive decompression reducing the ISP, resulting in recovery of the spinal cord blood supply and electrophysiological abnormalities (22).

Compared with laminectomy in previous studies (9, 11), similar clinical improvements can be achieved with extensive laminoplasty, as in the present study (38). However, because the laminae are still available for load bearing and attachment of the paraspinous muscles, laminoplasty can reduce the risk of spinal segment deterioration, daily microtrauma, instability, late kyphosis, and neurological deficits (induced by postlaminectomy membrane) (38–40). In this study, open-door laminoplasty was implemented because of its low surgical difficulty, short time, limited trauma and enough spinal canal area compared with double-door laminoplasty (41). At least one year of follow-up, only two cases cervical kyphosis were found; this low incidence may be related to the routinely protection of the posterior cervical muscles and reconstruction the C2 extensor attachment points (42, 43). In addition, due to the retention of lamina medial cortex on the hinge side and the use of titanium plates, no reclosed open-door was observed during follow-up.

Finally, we acknowledge that this study has inherent limitations as a retrospective, single-center investigation. The sample size and the lack of a comparison between surgery with anterior and combined posterior and anterior approaches are also limitations of this study. Furthermore, in patients with intramedullary hematoma and edema signals in long segments, we did not perform hematoma elimination or duraplasty. Previously several investigations have indicated that even laminectomy may not sufficiently improve the ISP and attenuate SCHS and that duraplasty may have to be required in severe tcSCI patients (9, 12, 44), as is practiced in decompressive craniectomy for diffuse traumatic brain injury (45–47). Therefore, further research is required to explore whether supplemental duraplasty and intramedullary hematoma elimination can further promote spinal cord function recovery compared with stand-alone extensive laminoplasty.

## Conclusion

Our retrospective analysis results suggest that early extensive open-door laminoplasty promotes neurological recovery by promoting the rapid resolution of spinal cord edema. The recovery of spinal function was more significant in patients with CSS. Larger, prospective controlled studies are needed to validate these findings, and duraplasty and intramedullary hematoma elimination will be further explored.

## Data Availability

The raw data supporting the conclusions of this article will be made available by the authors, without undue reservation.
